# Evaluation of the Nanodomain Structure in In-Zn-O Transparent Conductors

**DOI:** 10.3390/nano11010198

**Published:** 2021-01-14

**Authors:** Javier García-Fernández, Almudena Torres-Pardo, Julio Ramírez-Castellanos, Marta D. Rossell, José M. González-Calbet

**Affiliations:** 1 Inorganic Chemistry Department, Chemical Sciences Faculty, Universidad Complutense de Madrid, 28040 Madrid, Spain; javgar07@ucm.es (J.G.-F.); atorresp@ucm.es (A.T.-P.); jrcastel@ucm.es (J.R.-C.); 2 Electron Microscopy Center, Empa, Swiss Federal Laboratories for Materials Science and Technology, 8600 Dübendorf, Switzerland; marta.rossell@empa.ch; 3 ICTS National Center for Electron Microscopy, Universidad Complutense de Madrid, 28040 Madrid, Spain

**Keywords:** nano-characterization, (Cs)-corrected electron microscopy, geometric phase analysis, Zn_k_In_2_O_k+3_ homologous series

## Abstract

The optimization of novel transparent conductive oxides (TCOs) implies a better understanding of the role that the dopant plays on the optoelectronic properties of these materials. In this work, we perform a systematic study of the homologous series Zn_k_In_2_O_k+3_ (IZO) by characterizing the specific location of indium in the structure that leads to a nanodomain framework to release structural strain. Through a systematic study of different terms of the series, we have been able to observe the influence of the *k* value in the nano-structural features of this homologous series. The stabilization and visualization of the structural modulation as a function of *k* is discussed, even in the lowest term of the series (*k* = 3). The strain fields and atomic displacements in the wurtzite structure as a consequence of the introduction of In^3+^ are evaluated.

## 1. Introduction

Transparent conductive oxides (TCOs) are a family of functional materials that have received considerable attention in the last few years due to their potential optoelectronic applications. Among their main advantages, TCOs have awakened attention because these semiconductor oxides have an energy band gap between 3 and 5 eV combined with a relatively high electrical conduction, due to the presence of point defects [1]. These qualities make TCOs both, conductors and transparent to visible light. Some of the most studied oxides in this family of materials are In_2_O_3_, SnO_2_, Ga_2_O_3_, ZnO, among others. However, recent studies warn about the use of critical elements like In, Ga, Ge, etc., in TCOs attending to economic reasons and supply risk in optoelectronic devices [2]. Therefore, in order to reduce the amount of the crucial elements while maintaining their functionality, the search for novel materials goes through the synthesis of superstructures resulting from the intergrowth of two structural types. In this sense, the homologous series of indium zinc oxides (IZO), with chemical formula Zn_k_In_2_O_k+3_, keeps its conductivity values and high transmittance in the visible region [3] while satisfying the requirements for decreasing indium content with respect to the binary oxides and also others ternary oxides, like the well-known In-Sn-O (ITO). Additionally, the tunable electrical, photocatalytic, thermoelectric and luminescent properties as a function of the ZnO content (*k* value) widen the potential applications of the IZOs series as TCOs [3,4,5,6].

**Figure 1:**
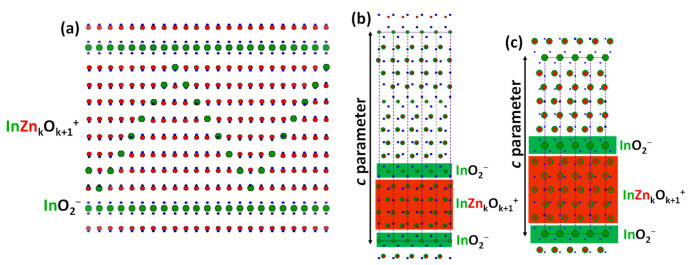
(**a**) Schematic structural model for *k* = 7 along the [1$\bar{1}$0] direction. Schematic structural model for (**b**) *k* = 3 and (**c**) *k* = 4 along the [010] direction. Indium, zinc and oxygen atoms are represented in green, red, and blue, respectively.

The structure of IZOs consists of an ordered intergrowth of InO_2_^-^ layers in octahedral coordination (hereinafter referred to as In–O layers) and wurtzite-like InZn_k_O_k+1_^+^ blocks, where indium and zinc occupy trigonal bipyramidal and tetrahedral sites, respectively, (for the sake of simplicity referred to as In/Zn–O blocks) stacked along the *c*-axis (Figure 1a). The symmetry of the homologous series Zn_k_In_2_O_k+3_ can be described according to the *R*-3*m* spatial group for *k* odd values, while *k* even terms can be characterized with the spatial group *P*6_3_/*mm* [7,8]. This is because, in odd *k* terms, the *c* parameter comprises the distance between 3 consecutive In-O layers, whereas, in *k* even members only, 2 consecutive In-O layers are included. Figure 1b,c shows the schematic structural representation of *k* = 3 and *k* = 4 members along the [010] direction, where the *c* parameter is indicated. For terms with *k* ≥ 6 a modulation showing a zig-zag or a wave-like shape has been observed by transmission electron microscopy (TEM) [9,10,11]. This modulation consists of an inhomogeneous distribution of the In^3+^ nanoclusters inside the wurtzite blocks following a zig-zag pattern in order to minimize the internal deformation produced by the different ionic radii of In^3+^ (0.80 Å) and Zn^2+^ (0.60 Å) [12]. The modulation for terms with *k* < 6 has been more recently identified, although no systematic studied has been performed to date [6]. In summary, IZOs are emerging as commercially viable transparent conducting oxides because of its excellent optical transmission, good electrical conductivity and high chemical and thermal stability, whose characteristic atomic distribution plays an important role in the electronic properties [13,14].

The origin of the zig-zag modulation on IZO nanowires was studied by Goldstein et al. [15] using spherical aberration (Cs)-corrected microscopy by scanning TEM mode and density functional theory (DFT) calculations. These authors suggested that the zig-zag of In^3+^ arises from an inversion of the metal and oxygen positions on both sides of the In^3+^ trigonal bipyramidal positions within the wurtzite block. Their model reveals that this defect runs along the {1-21*l*} planes of the wurtzite structure, being the most stable computed value *l* = 3. According to their results, the calculated angle formed by the intersection of the zig-zag plane and the In-O layer is 46.8°. Nevertheless, the nature of this modulation has been the subject of intense debate during the last years [15,16,17,18]. Indeed, previous studies showed a clear influence of the nature of M^3+^ on the modulation. Actually, when In^3+^ is replaced by Fe^3+^ the angle and periodicity of the zig-zag is modified [11,15,16,17,18], and when In^3+^ is replaced by Ga^3+^ or Al^3+^, the zig-zag modulation disappears, placing both cations in the middle of the block [19]. However, there are not details about the influence of the In^3+^ distribution in the wurtzite-block as a function of the *k* term and most notable is the lack of information on the lower members of this homologous series. In this work, we focus on the effect that the zig-zag distribution of In^3+^ causes on the wurtzite-like InZn_k_O_k+1_^+^ blocks as a function of the *k* term, with special attention to those terms with a low *k* value. For this propose, we have carried out an exhaustive study of the In^3+^ modulation in three different terms of the series (*k* = 3, 7 and 11) and we have evaluated the lattice deformation caused by the In^3+^ within the wurtzite-type block by exploiting the capabilities offered by aberration-corrected (S)TEM operating at low electron dose to prevent In^3+^ from diffusing.

## 2. Materials and Methods

The three terms of the homologous series Zn_k_In_2_O_k+3_ (*k* = 3, 7, and 11) were obtained by conventional solid state reactions. A mixture of appropriate amounts of the constituent oxides ZnO (Aldrich, St. Louis, Missouri, USA, 99.99%) and In_2_O_3_ (Aldrich, St. Louis, Missouri, USA, 99.99%) was treated under different thermal conditions depending on the *k* value in order to avoid the formation of impurities and disordered intergrowths of different terms. In this regard, we prepared all samples at 1350 °C and 56 h for *k* = 3, 48 h for *k* = 7, and 24 h for *k* = 11. These temperatures and reaction times have been used to obtain single-phases and well-ordered materials, according to the phase diagram published by Moriga et al. [3].

X-ray diffraction (XRD) patterns were recorded using a Panalytical X’Pert Pro Alpha1 instrument (Malvern Panalytical, Malvern, UK), equipped with a primary fast X’Celerator detector operating at 45 kV and 40 mA, and fitted with a primary curved Ge (111) monochromator in order to obtain Cu Kα_1_ radiation (λ = 1.5406 Å). Data were collected at 2θ between 5° and 70°, with a step size of 0.02° and a collection time of 3 s per step.

Conventional High Resolution Transmission Electron Microscopy (HRTEM) and Selected Area Electron Diffraction (SAED) data were acquired in a JEOL JEM 3000F (JEOL, Tokyo, Japan) operated at 300 kV. The (Cs)-corrected HRTEM images were obtained using a JEOL JEM GRAND ARM 300cF microscope (JEOL, Tokyo, Japan) equipped with a Cs corrector (ETA-JEOL). A precise measurement of aberrations and an optimized correction were achieved using the JEOL COSMO corrector control software (JEOL, Tokyo, Japan). The accelerating voltage was set to 120 kV. HRTEM images were acquired using a Gatan OneView CMOS camera (Gatan Inc., Pleasanton, USA) (4096 × 4096 pixels). The Scanning Transmission Electron Microscopy (STEM) experiments were carried out on an aberration-corrected Titan Themis 80–300 (Thermo Fisher Scientific, Eindhoven, Netherlands) equipped with a probe CEOS DCOR spherical aberration corrector (CEOS GmbH, Heidelberg, Germany) operated at 300 kV, setting a probe semi-convergence angle of 18 mrad and collecting semi-angles of 90–170 mrad for high-angle annular dark-field (HAADF) imaging. The HAADF images were obtained through aligning and averaging series of 10 short-exposure images (1 μs dwell time, 2048 × 2048) by means of the SmartAlign [20] software (version 2.5, HREM Research Inc., Tokyo, Japan), in order to increase the signal-to-noise ratio and to correct for scan distortions. Geometric Phase Analysis (GPA) of the resulting distortion-corrected images was performed by using the GPA tool contained in the FRWRtools plugin (Humboldt-Universität zu Berlin, Berlin, Germany) [21] for Gatan Digital Micrograph (version 1.85, Gatan Inc., Pleasanton, CA, USA). The g(001) and g(110) Bragg spots in the Fourier transform of the lattice images were used for analysis with the following parameters: resolution = 0.6 nm, smoothing = 5.0. Additionally, the positions of the atomic columns were directly determined on the deconvoluted images (maximum entropy method, calculated probe 50 pm, 80 iterations) by using the STEM_CELL software package (version 2.5, CNR-Istituto Nanoscienze, Modena, Italy) [22] and by subsequently performing an iterative refining of the fitted peaks solving a least-squares minimization problem (using the Levenberg–Marquardt algorithm). This refinement was carried out using a custom-developed script that makes use of 7-parameter two-dimensional Gaussians. The fitting allows a quantitative estimation of the atomic column peak intensities and their positions with picometer precision [23]. After fitting the atomic column peaks, the atomic displacements along the [001] direction of the Zn/In cations were measured relative to the center of each atomic row running along [110] by using a custom-developed algorithm in MATLAB R2019b (version 9.7, MathWorks, Natick, MA, USA). The vectors in the arrow maps are plotted along the displacement direction of the Zn/In cations, that is, away from the oxygen columns. For electron microscopy observations, the samples were ground in an agate mortar, ultrasonically dispersed in n-butanol, and transferred to carbon-coated copper grids.

## 3. Results and Discussion

**Figure 2:**
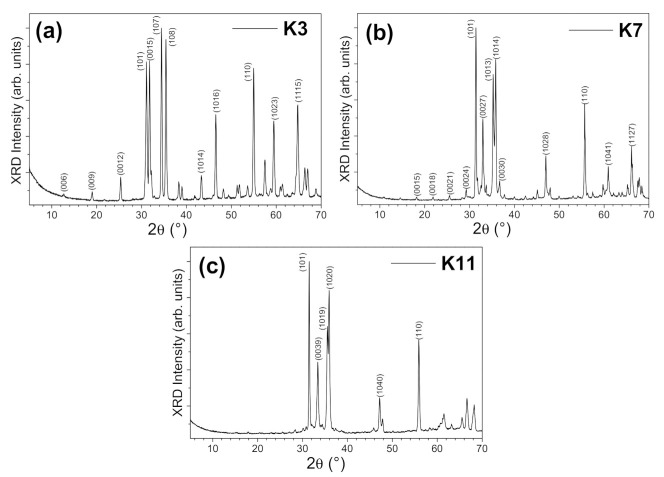
XRD patterns of the prepared IZO samples. (**a**) *k* = 3, (**b**) *k* = 7, and (**c**) *k* = 11. The most intense diffraction maxima have been indexed.

Figure 2a,c display the X-ray powder diffraction (XRD) patterns of the as-prepared samples Zn_3_In_2_O_6_ (*k* = 3), Zn_7_In_2_O_10_ (*k* = 7), and Zn_11_In_2_O_14_ (*k* = 11). All samples can be indexed on the basis of a *R*-3*m* space group and the obtained cell parameters for each term are collected in Table 1, in agreement with previously reported data [3,7,8].

**Figure 3:**
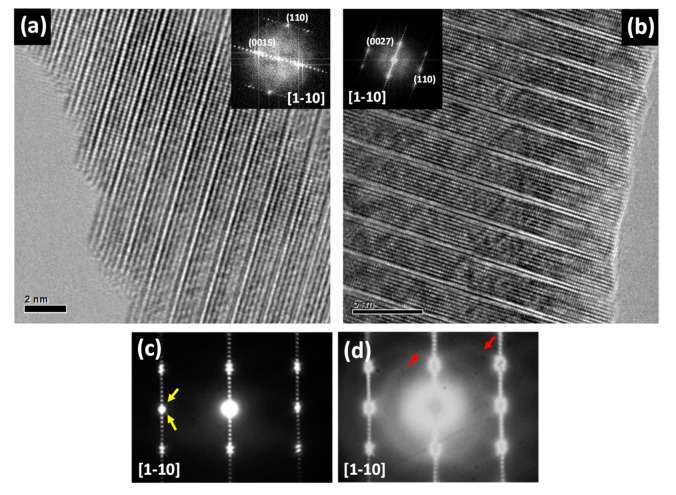
HRTEM images of (**a**) *k* = 3 and (**b**) *k* = 7 along the [1$\bar{1}$0] direction. The inset shows the corresponding FFT. (**c**) SAED pattern of *k* = 7 sample recorded under typical density current of around 10^−8^ A·cm^−2^ with 2 s exposure time. (**d**) SAED pattern of *k* = 7 sample recorded under higher brightness with 16 s exposure time.

Figure 3a,b show representative conventional HRTEM images along the [1$\bar{1}$0] zone axis and their corresponding Fast Fourier Transforms (FFT) (insets) of the terms *k* = 3 and 7. Both images clearly show the formation of the superstructure and the stacking of the wurtzite blocks along the *c* direction in a clearly ordered way. Although for *k* = 3 a subtle variation in contrast is observed in the wurtzite block, this feature is unambiguously observed for *k* = 7 as a dark zig-zag contrast inside the wurtzite block. As we discussed above, this contrast is attributed to the structural modulation formed by the inhomogeneous distribution of In^3+^ in nanoclusters for terms *k* ≥ 6 [9,10,11]. This is confirmed by the presence of satellite reflections (marked with yellow arrows), which are clearly visualized in the corresponding SAED pattern (Figure 3c) for the *k* = 7 term. It is worth mentioning that, when the SAED pattern is recorded under high brightness and high exposure times (Figure 3d), some diffuse lines (indicated by red arrows for better visualization) appear crossing the diffraction pattern, suggesting a structural order involving different wurtzite blocks.

At this point it is worth mentioning that, In^3+^ can diffuse under the electron beam making more challenging the interpretation of the experimental data. In order to assess the character of these extra inter-block order while assuring the integrity of the samples, the different IZO terms were investigated by low-dose (Cs)-corrected HRTEM by using a high sensitivity camera. In this sense, we studied the materials in milder working conditions in terms of radiation by the electron beam, avoiding the diffusion of In^3+^ cations that would result on non-real data.

**Figure 4:**
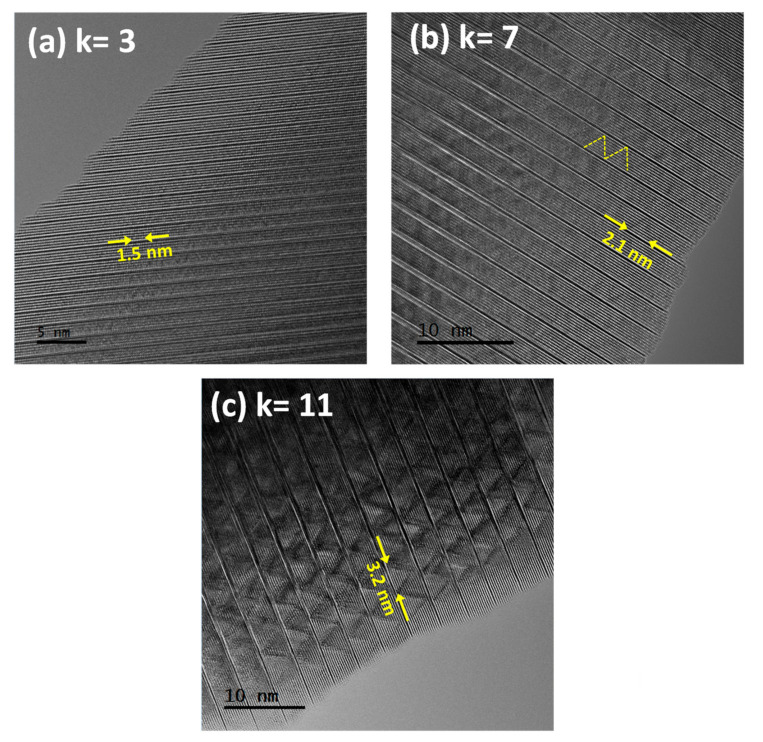
HRTEM images corresponding to (**a**) *k* = 3, (**b**) *k* = 7, and (**c**) *k* = 11 along the [1$\bar{1}$0] direction. Dashed yellow lines in the *k* = 7 term are marked for a better visualization of the zig-zag pattern. In each image, the periodicity of the modulation is indicated.

Figure 4a,c show the HRTEM images along the [1$\bar{1}$0] direction for the three studied samples. The zig-zag contrast is identified within the wurtzite block. Notice that this contrast is also observed in the lowest term of the series (*k* = 3), although the contrast of the zig-zag modulation is enhanced with increasing *k*. This fact unambiguously confirms that the inhomogeneous distribution of In^3+^ along the zig-zag planes is an inherent characteristic of all the terms of the homologous series as opposed to the generally accepted description that the modulation would only occur for terms with *k* ≥ 6 [9,10,11].

**Figure 5:**
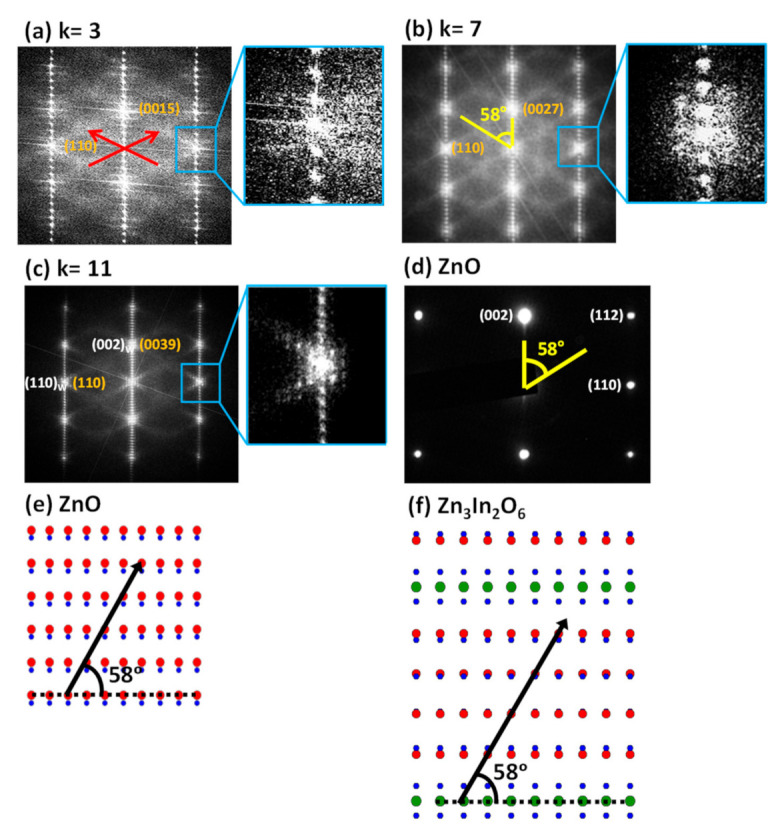
Fourier Transforms of HRTEM images corresponding to the (**a**) *k* = 3, (**b**) *k* = 7, and (**c**) *k* = 11 terms and enlarged area around the (110) reflection as indicated by the blue boxes. In *k* = 3, the direction of the diffuse lines is indicated with red arrows. In *k* = 7, the angle between the diffuse lines and the (00*l*) planes, 58°, is shown. In *k* = 11, the reflections belonging to the basic substructure (wurtzite) are indicated in white. (**d**) SAED pattern corresponding to ZnO along [1$\bar{1}$0]. (**e**) and (**f**) Representative schemes of ZnO and Zn_3_In_2_O_6_ along [1$\bar{1}$ 0]. The arrows indicate the direction of the modulation and the dashed lines the (00*l*) planes. Color code: In (green), Zn (red), O (blue).

The corresponding FFTs of the described images are displayed in Figure 5a–c. The presence of satellite reflections in all terms is confirmed (see the enlarged area around the (110) reflection) although, the definition of these maxima is consolidated for the higher *k* terms, as expected. Note that the angle formed by satellite reflections and the (00*l*) planes remains constant for all the terms with a value of 58°, revealing that the In^3+^ is always distributed along the [112] direction of the ZnO wurtzite basic structure regardless of the thickness of the wurtzite-type In/Zn–O block in each term of the homologous series (Figure 5d). Therefore, the In^3+^ distribution within the wurtzite-type blocks seems to be independent of the *k* value and, as a result, the period of the zig-zag modulation varies with *k,* such that period values of 1.5, 2.1, and 3.2 nm were obtained for the terms *k* = 3, 7, and 11, respectively (see Figure 4).

The high sensibility of the camera also allowed us to detect, for all the samples, the presence of diffuse, but clearly directional, lines (marked, as example, with red lines in Figure 5a) along the [112] direction of the wurtzite-basic structure, the same crystallographic directions observed for the zig-zag satellite spots. Therefore, the diffuse lines indicate that there is a short-range order correlation between the zig-zag planes of In^3+^ along the different wurtzite blocks. Effectively, as it can be inferred from the images in Figure 4, the In^3+^ distribution is not perfectly ordered between the blocks, but shows a tendency to correlate along the [112] directions of the wurtzite-basic structure resulting in an inhomogeneous distribution of In^3+^ nanoclusters in the structure. In the homologous series, this direction takes a different value for each term due to the different value of *l*. Thus, it would be the [1115] direction for the term *k* = 3, [1127] for *k* = 7, and [1139] for *k* = 11. Therefore, the direction of these diffuse lines appears in a general way, along the [113*k*+6] direction for the odd *k* terms and [112*k*+4] for the even *k* terms. This difference has its origin in the different spatial groups used to describe the odd and even *k* terms of the homologous series, as discussed earlier in the introduction. A schematic representation of ZnO and Zn_3_In_2_O_6_ (*k* = 3) along [1$\bar{1}$0] is shown in Figure 5e,f, respectively. In this representation, the ZnO [112] direction and the corresponding modulation direction in the term *k* = 3, that is, the [1115], have been indicated with black arrows, and are displayed together with the (00*l*) planes in dashed lines and the 58° angle resulting from the intersection with the modulation direction.

It is important to note that the In/Zn ratio within the wurtzite block decreases as *k* increases, although the location of In^3+^ along the [112] wurtzite direction appears to be independent of the *k* value. In this sense, those terms with lower *k* values must accommodate more In^3+^ in a smaller number of atomic planes within the InZn_k_O_k+1_^+^ blocks as compared with the higher *k* terms. Therefore, it is expected that the In^3+^ located within the wurtzite-type blocks will result in distinct strain modulations for the different *k* terms of the homologous series.

**Figure 6:**
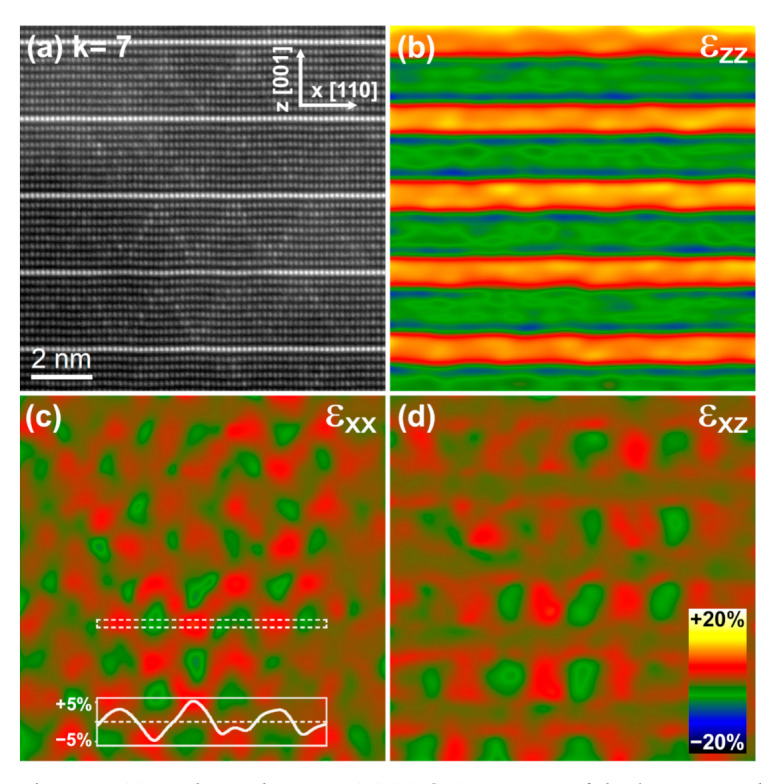
(**a**) High-resolution HAADF-STEM image of the *k* = 7 term obtained from aligning and averaging a series of 10 short-exposure images. Note the presence of the zig-zag modulation with a brighter contrast in the wurtzite-type blocs. (**b**–**d**) Corresponding ε_zz_, ε_xx_, and ε_xz_ strain maps obtained by GPA. The inset in panel (**c**) is the line profile of the ε_xx_ strain component extracted along the white dashed box. The color bar gives the change in strain.

**Figure 7:**
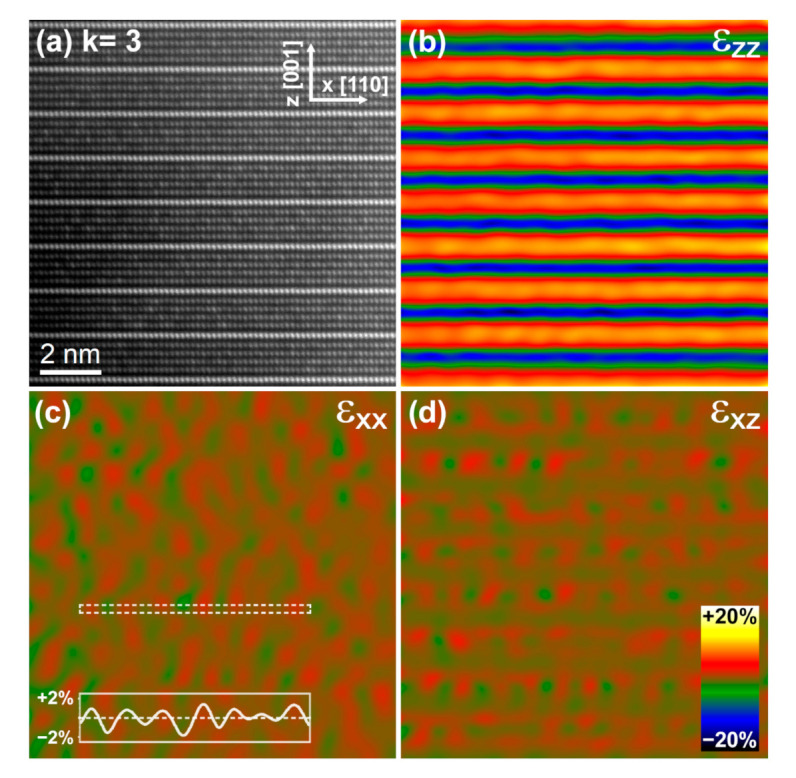
(**a**) High-resolution HAADF-STEM image of the *k* = 3 term obtained from aligning and averaging a series of 10 short-exposure images. The zig-zag modulation is barely visible. (**b**–**d**) Corresponding ε_zz_, ε_xx_, and ε_xz_ strain maps obtained by GPA. The inset in panel (**c**) is the line profile of the ε_xx_ strain component extracted along the white dashed box. The color bar gives the change in strain.

In the following, we evaluate the deformation fields for the *k* = 3 and *k* = 7 terms, that is, below and above *k* = 6, the term that has so far been recognised as the lowest in the Zn_k_In_2_O_k+3_ homologous series displaying the zig-zag structural modulation [9,10,11]. For this purpose, HAADF-STEM images were obtained through aligning and averaging HAADF-STEM image series in order to correct for scan distortions, see Figure 6a and Figure 7a. Geometric Phase Analysis (GPA) [24] was applied to measure the local deformations of the atomic lattice from the distortion-corrected HAADF-STEM images. The obtained local strain components are displayed as color-coded maps in Figure 6b–d and Figure 7b–d. As expected, the ε_zz_ strain component of Figure 6b and Figure 7b provides evidence for large lattice dilations along the [001] direction around the octahedral In–O layers. The dilation at these positions as compared to the wurtzite-type blocs is about 15 and 22% for the *k* = 7 and 3, respectively. This observation is in good agreement with the *c* lattice parameter values obtained by XRD. More interesting are, however, the strain features observed in the ε_xx_ strain map of Figure 6c. Here, large dilations (in red) coincide with the In^3+^ trigonal bipyramidal positions conforming the zig-zag structures present in the wurtzite-type blocks. Moreover, it is seen that the dilations are clearly larger at the apices of the zig-zag features than at the positions connecting neighboring apices. A line profile extracted along the white dashed box is shown as an inset in Figure 6c. The dilatation strain at the apices of the zig-zag (in red) is up to 10% larger than at the center of the domains (in green). This value is more than twofold higher than the 4.5% value reported for the *k* = 30 term [25]. In Figure 7c a similar strain pattern with maxima and minima is observed for the *k* = 3 term. However, here the alternation of maxima and minima appears more disorganized and clear zig-zag features are only locally identified (see, e.g., the upper left corner). The line profile extracted along the white dashed box in Figure 7c reveals that the strain difference between the (red) zig-zag apices and the (green) domains is about 3%, revealing a more homogeneous distribution of the In cations in the wurtzite-type layers than in the *k* = 7 term. Finally, the ε_xz_ strain maps of both terms displayed in Figure 6d and Figure 7d exhibit very similar strain modulations further supporting the presence of a structural modulation in the *k* = 3 term.

Interestingly, in Figure 6a, subtle Zn/In atomic column displacements along the *c* axis are clearly perceived when crossing the zig-zag features. These shifts were previously reported to be the result of a change in the orientation of the ZnO_4_ tetrahedra on both sides of the zig-zags [15,19,26]. Hence, the zig-zag features are inversion domains boundaries (IDBs) that separate adjacent domains of opposite polarity. We next investigated the inversion domain boundary distribution and the magnitude of the Zn/In atomic column displacements in the *k* = 7 and 3 terms.

**Figure 8:**
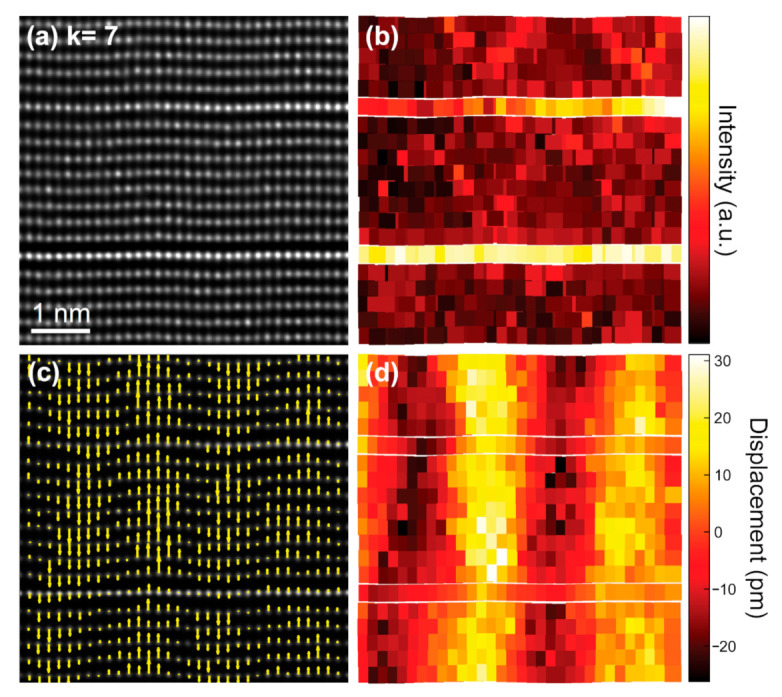
(**a**) Deconvoluted HAADF-STEM image of the *k* = 7 term revealing the undulation of the rows of Zn/In atomic columns. (**b**) Intensity map of the Zn/In atomic columns as obtained from the HAADF signal. (**c**) Atomic displacements along the (vertical) [001] direction of the Zn/In atomic columns measured relative to the center of each atomic row. The vectors in the arrow map are plotted along the displacement direction of the Zn/In cations, i.e., away from the oxygen columns. (**d**) Extracted fit of the atomic displacements for the Zn/In atomic columns plotted at their fitted coordinates.

**Figure 9:**
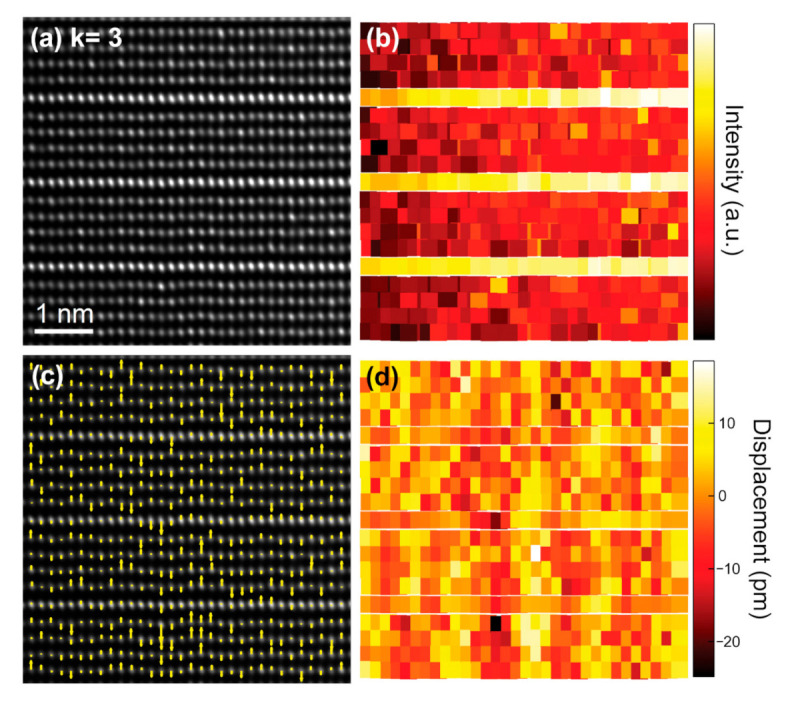
(**a**) Deconvoluted HAADF-STEM image of the *k* = 3 term revealing the undulation of the rows of Zn/In atomic columns. (**b**) Intensity map of the Zn/In atomic columns as obtained from the HAADF signal. (**c**) Atomic displacements along the (vertical) [001] direction of the Zn/In atomic columns measured relative to the center of each atomic row. The vectors in the arrow map are plotted along the displacement direction of the Zn/In cations, i.e., away from the oxygen columns. (**d**) Extracted fit of the atomic displacements for the Zn/In atomic columns plotted at their fitted coordinates.

The positions of the atomic columns were directly determined on the deconvoluted images shown in Figure 8a and Figure 9a. Our fitting allows a quantitative estimation of the atomic column peak intensities and their positions with a precision of about 3 pm (see Materials and Methods section for details). The corresponding intensity maps of the metal sublattice are depicted in Figure 8b and Figure 9b. The zig-zag features are clearly visible with a brighter contrast in the intensity map of the *k* = 7 term (Figure 8b), but are only distinguishable in the lower left side of the intensity map of the *k* = 3 term (Figure 9b). Subsequently, the atomic displacements along the [001] direction of the Zn/In cations were measured relative to the center of each atomic row running along the [110] direction. The resulting vector maps are presented in Figure 8c and Figure 9c. The vectors in the arrow maps are plotted along the displacement direction of the Zn/In atomic columns, that is, away from the oxygen columns (not visible in the HAADF images). In both terms, it is evident that adjacent domains exhibit opposite polarity. As well, the Zn/In atomic column displacements are larger in the center of the domains and gradually diminish towards the IDBs. Figure 8d and Figure 9d are scattered color-code plots that give the magnitude of the Zn/In atomic column displacements in picometers. In the vicinity of the zig-zag apices, the measured atomic displacements are maximum; they are of the order of ±25 and ±13 pm for the *k* = 7 and 3 terms, respectively.

Similarly to the strain analysis by GPA, the displacement analysis of the Zn/In atomic columns supports the occurrence of a zig-zag structural modulation in the *k* = 3 term of the Zn_k_In_2_O_k+3_ homologous series. Nevertheless, compared to the *k* = 7 term, in the *k* = 3 term the modulation is less defined and only locally visible. This would explain the lower definition of the satellite reflections observed in the FFTs of Figure 5a.

Considering the results shown above, an unambiguous nanodomain structure is identified for the Zn_k_In_2_O_k+3_ series whose origin is related with the zig-zag distribution of indium along the [113*k*+6] direction for the odd *k* terms and [112*k*+4] for the even *k* terms within the wurtzite block. The indium-induced structural distortion in the wurtzite block results in a change in the orientation of the ZnO_4_ tetrahedra on both sides of the indium planes (IDBs), giving rise to the nanodomain framework. The evaluation of atomically resolved vector maps also reveals that the nanodomains tend to correlate between different wurtzite-type blocks along the *c* direction, proving that the indium distribution has a significant effect on the entire structure of these materials. Interestingly, the indium distribution has revealed to be inherent to the homologous series, irrespective of the value of the *k* term. In this sense, a predictable behavior from the structural point of view can be expected for the Zn_k_In_2_O_k+3_ series in which a similar nanostructure framework must occur for all terms. Unfortunately, the fact of working with powdered samples rather than thin films, as well as the synthesis conditions (high temperature and long annealing times) could limit the potential use of these materials as TCOs. On the other hand, the possibility of perfectly controlling the In/Zn ratio from the *k* = 3 to the *k* = 11 expands the ability to tune the electro-optic functional response of the system, both characteristics confirming the high potential of IZOs for functional applications. The future work and perspectives of these phases would involve the development of new soft synthesis methods which allow the reduction of the particle size, and the possibility of integrating and growing these phases on substrates in a controlled way.

## Figures and Tables

**Table 1:** Lattice parameters obtained by XRD for *k* = 3, 7, and 11 terms.

*k*	a (Å)	c (Å)
3	3.35(1)	42.52(3)
7	3.31(1)	73.62(3)
11	3.28(1)	105.5(1)
